# A guiding framework for needs assessment evaluations to embed digital platforms in partnership with Indigenous communities

**DOI:** 10.1371/journal.pone.0279282

**Published:** 2022-12-22

**Authors:** Jasmin Bhawra, M. Claire Buchan, Brenda Green, Kelly Skinner, Tarun Reddy Katapally

**Affiliations:** 1 School of Occupational and Public Health, Toronto Metropolitan University, Toronto, ON, Canada; 2 School of Public Health Sciences, University of Waterloo, Waterloo, ON, Canada; 3 Île-à-la-Crosse School Division, The Northern Village of Île-à-la-Crosse, Île-à-la-Crosse, SK, Canada; 4 DEPtH Lab, Faculty of Health Sciences, Western University, London, ON, Canada; 5 Department of Epidemiology and Biostatistics, Schulich School of Medicine and Dentistry, Western University, London, ON, Canada; 6 Lawson Health Research Institute, London, Ontario, Canada; Swinburne University of Technology, AUSTRALIA

## Abstract

**Introduction:**

In community-based research projects, needs assessments are one of the first steps to identify community priorities. Access-related issues often pose significant barriers to participation in research and evaluation for rural and remote communities, particularly Indigenous communities, which also have a complex relationship with academia due to a history of exploitation. To bridge this gap, work with Indigenous communities requires consistent and meaningful engagement. The prominence of digital devices (i.e., smartphones) offers an unparalleled opportunity for ethical and equitable engagement between researchers and communities across jurisdictions, particularly in remote communities.

**Methods:**

This paper presents a framework to guide needs assessments which embed digital platforms in partnership with Indigenous communities. Guided by this framework, a qualitative needs assessment was conducted with a subarctic Métis community in Saskatchewan, Canada. This project is governed by an Advisory Council comprised of Knowledge Keepers, Elders, and youth in the community. An environmental scan of relevant programs, three key informant interviews, and two focus groups (n = 4 in each) were conducted to systematically identify community priorities.

**Results:**

Through discussions with the community, four priorities were identified: (1) the Coronavirus pandemic, (2) climate change impacts on the environment, (3) mental health and wellbeing, and (4) food security and sovereignty. Given the timing of the needs assessment, the community identified the Coronavirus pandemic as a key priority requiring digital initiatives.

**Conclusion:**

Recommendations for community-based needs assessments to conceptualize and implement digital infrastructure are put forward, with an emphasis on self-governance and data sovereignty.

## Introduction

Community engagement has been the cornerstone of participatory action research in a range of disciplines. Every community has a unique culture and identity, hence community members are the experts regarding their diverse histories, priorities, and growth [1–3]. As a result, the successful uptake, implementation, and longevity of community-based research initiatives largely depends on meaningful community engagement [4–9]. There is a considerable body of evidence establishing the need for ethical community-research partnerships which empower citizens and ensure relevant and sustainable solutions [1–3, 10]. For groups that have been marginalized or disadvantaged, community-engaged research that prioritizes citizens’ control in the research process can provide a platform to amplify citizens’ voices and ensure necessary representation in decision-making [11]. Such initiatives must be developed in alignment with a community’s cultural framework, expectations, and vision [12] to support continuous and meaningful engagement throughout the project. In particular, when partnering with Indigenous communities, a Two-Eyed Seeing approach can provide valuable perspective to combine the strengths of Indigenous and Western Knowledges, including culturally relevant methods, technologies, and tools [13–15].

Many communities have a complicated relationship with research as a result of colonialism, and the trauma of exploitation and discrimination has continued to limit the participation of some communities in academic partnerships [16]. Indigenous Peoples in Canada experience a disproportionate number of health, economic, and social inequalities compared to non-Indigenous Canadians [17]. Many of these health (e.g., elevated risk of chronic and communicable diseases) [18–21]), socioeconomic (e.g., elevated levels of unemployment and poverty) [19, 22–24], and social (e.g., racism and discrimination) [19, 22–24]) inequities can be traced back to the long-term impacts of assimilation, colonization, residential schools, and a lack of access to healthcare [19, 20, 22–24]. To bridge this gap, and more importantly, to work towards Truth and Reconciliation [25], work with Indigenous Peoples must be community-driven, and community-academia relationship building is essential before exploring co-conceptualization of initiatives [26].

One of the first steps in building a relationship is to learn more about community priorities by conducting a needs assessment [27, 28]. A needs assessment is a research and evaluation method for identifying areas for improvement or gaps in current policies, programs, and services [29]. When conducted in partnership with a specific community, needs assessments can identify priorities and be used to develop innovative solutions, while leveraging the existing knowledge and systems that communities have in place [30]. Needs assessments pave the path for understanding the value and applicability of research for community members, incorporating key perspectives, and building authentic partnerships with communities to support effective translation of research into practice.

For rural, remote, and northern communities within Canada, issues related to access (e.g., geographic location, transportation, methods of communication, etc.) pose significant barriers to participation in research and related initiatives [31]. Digital devices, and in particular, the extensive usage of smartphones [32] offers a new opportunity to ethically and equitably engage citizens [33]. Digital platforms (also referred to as digital tools) are applications and software programs accessible through digital devices. Digital platforms can be used for a variety of purposes, ranging from project management, to healthcare delivery or mass communication [34]. Digital infrastructure–the larger systems which support access and use of these digital platforms, including internet, satellites, cellular networks, and data storage centres [34]. The Coronavirus (COVID-19) pandemic has catalyzed the expansion of digital technology, infrastructure and the use of digital devices in delivering essential services (e.g., healthcare) and programs to communities [35, 36].

While digital platforms have been used in Indigenous communities for numerous initiatives, including environmental mapping initiatives (e.g., research and monitoring, land use planning, and wildlife and harvest studies) [37, 38] and telehealth [39], there has largely been isolated app development without a corresponding investment in digital infrastructure. This approach limits the sustainability of digital initiatives, and importantly does not acknowledge an Indigenous world view of holistic solutions [39].

Thus given the increasing prominence of digital devices [39, 40], it is critical to evaluate the conceptualization, implementation, and knowledge dissemination of digital platforms. To date, there is little guidance on how to evaluate digital platforms, particularly in partnership with rural and remote communities [41]. A review of recent literature on community-based needs assessments uncovered numerous resources for conducting evaluations of digital platforms, however, a key gap is the lack of practical guidance for conducting needs assessments in close collaboration with communities in ways that acknowledge existing needs, resources, supports and infrastructure that also incorporates the potential role of digital platforms in addressing community priorities.

This paper aims to provide researchers and evaluators with a framework (step-by-step guide) to conduct needs assessments for digital platforms in collaboration with Indigenous communities. To achieve this goal, a novel needs assessment framework was developed using a Two-Eyed Seeing approach [13–15] to enable the identification of community priorities, barriers and supports, as well as existing digital infrastructure to successfully implement digital solutions. To demonstrate the application of this framework, a community-engaged needs assessment conducted with a subarctic Indigenous community in Canada is described and discussed in detail.

## Framework design and development

This project commenced with the design and development of a new framework to guide community-based needs assessments in the digital age.

### Needs assessments

Needs assessments are a type of formative evaluation and are often considered a form of strategic or program planning, even more than they are considered a type of evaluation. Needs assessments can occur both before and during an evaluation or program implementation; however, needs assessments are most effective when they are conducted before a new initiative begins or before a decision is made about what to do (e.g., how to make program changes) [29]. Typically, a needs assessment includes: 1) collecting information about a community; 2) determining what needs are already being met; and 3) determining what needs are not being met and what resources are available to meet those needs [42].

### Framework development

Based on existing literature, community consultation, and drawing expertise from our team of evaluation experts who have over a decade of experience working with Indigenous communities on a range of research and evaluation projects, a novel framework was developed to guide community-based needs assessments focused on the application of digital platforms.

**Fig 1:**
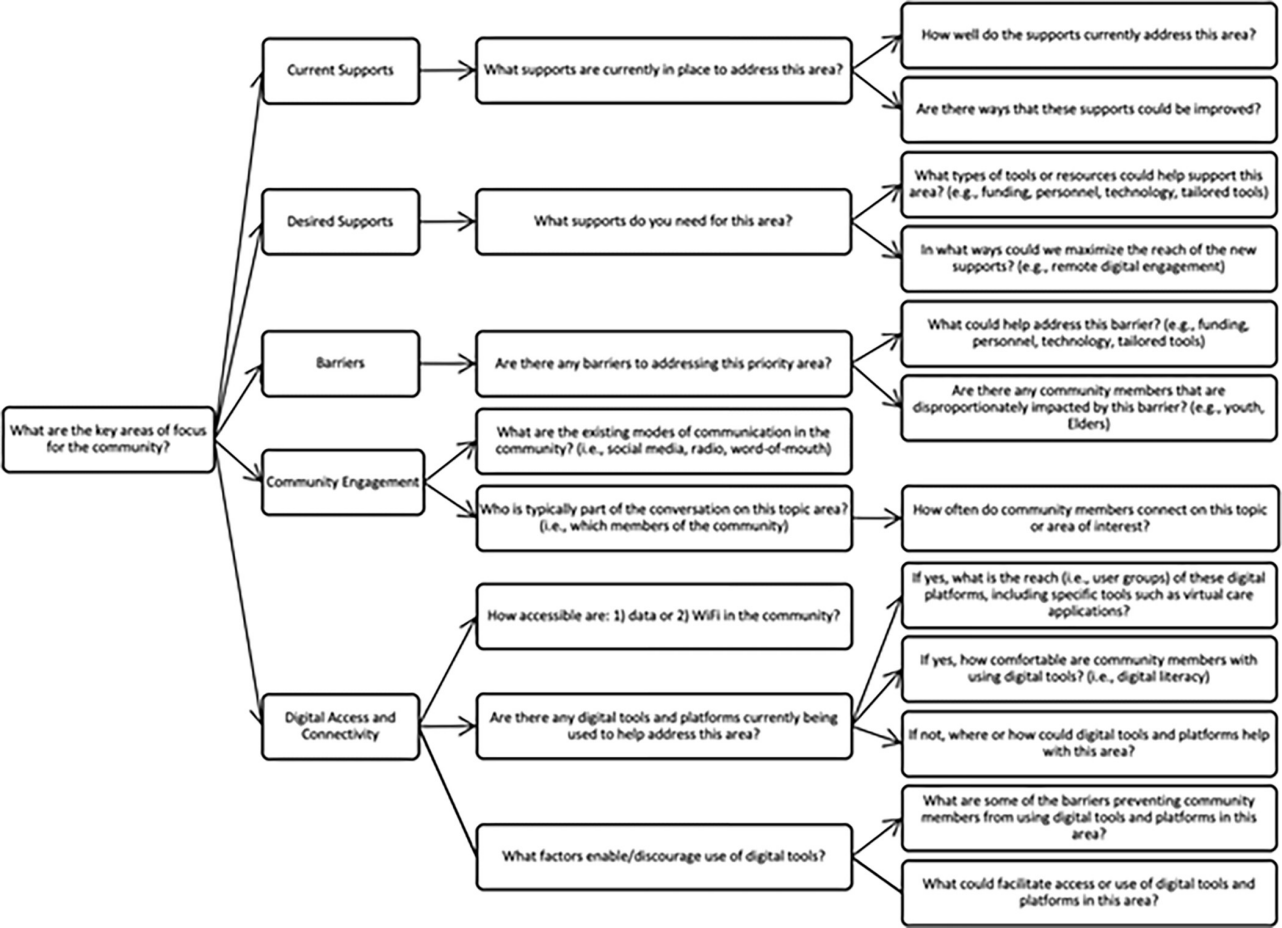
A guiding framework for community-based needs assessments to embed digital platforms.

This framework (see **Fig 1**) is driven by core questions necessary to identify community priorities that can be addressed by developing and implementing digital platforms. Through team discussion and community consultation, five key topic areas for the assessment of community needs were identified: i) current supports; ii) desired supports; iii) barriers; iv) community engagement; and v) digital access and connectivity. A series of general questions across the five needs assessment topic areas were developed. Thereafter, a set of sub-questions were embedded in each key topic area.

The Guiding Framework outlines an approach for conducting community needs assessments which can be adapted across communities and jurisdictions. This framework offers a flexible template that can be used iteratively and applied to various community-engaged needs assessments in a range of areas, including but not limited to community health and wellness projects. The questions assigned to each topic area can be used to guide needs assessments of any priority identified by community stakeholders as suitable for addressing with digital platforms.

## Needs assessment methods

The Guiding Framework was implemented in collaboration with a subarctic Indigenous community in Canada, and was used to identify key community priorities, barriers, supports, and existing digital infrastructure which could inform the design and implementation of tailored digital platforms.

### Design

Using an environmental scan of relevant documents and qualitative focus groups and interviews, a needs assessment was conducted with the Northern Village of Île-à-la-Crosse, Saskatchewan, Canada between February and May 2020.

This project is governed by a Citizen Scientist Advisory Council which included researchers, Knowledge Keepers, Elders, and youth from Île-à-la-Crosse. The study PI (TRK) and Co-Investigator (JB) developed a relationship with key decision-makers in Île-à-la-Crosse in 2020. Through their guidance and several community visits, the decision-makers introduced the research team to Elders, youth, and other community members to gain a better understanding of current priorities and needs in Île-à-la-Crosse. The research team developed relationships with these community members and invited them to join the Council to formally capture feedback and plan ongoing projects to promote health and wellbeing in the community. The Council represents the needs and interests of the community, and guides the project development, implementation, and evaluation. Council members were provided with Can $150 (US $119.30) as honoraria for each meeting to respect their time, knowledge, and contributions.

Written consent was obtained from all focus group participants and verbal consent was obtained from all key informants participating in interviews. This study received ethics clearance from the research ethics boards of the University of Regina and the University of Saskatchewan through a synchronized review protocol (REB# 2017–29).

### Setting

Established in 1776, Île-à-la-Crosse is a northern subarctic community with road access in northwest Saskatchewan. Sakitawak, the Cree name for Île-à-la-Crosse, means “where the rivers meet,” hence the community was an historically important meeting point for the fur trade in the 1800s [43, 44] The community lies on a peninsula on the Churchill River, near the intersections with the Beaver River and Canoe River systems. Île-à-la-Crosse has a rich history dating back to the fur trade. Due to its strategic location, Montreal-based fur traders established the first trading point in Île-à-la-Crosse in 1776, making the community Saskatchewan’s oldest continually inhabited community next to Cumberland House [45]. In 1821, Île-à-la-Crosse became the headquarters for the Hudson’s Bay Company’s operations in the territory. In 1860, the first convent was established bringing Western culture, medical services, and education to the community.

Île-à-la-Crosse has a population of roughly 1,300 people [19]. Consistent with Indigenous populations across Canada, the average age of the community is 32.7 years, roughly 10 years younger than the Canadian non-Indigenous average [19]. Census data report that just under half (44%) of the community’s population is under the age of 25, 46.3% are aged 25–64, and 9.3% aged 65 and over [19]. Members of the community predominantly identify as Métis (77%), with some identifying as First Nations (18%), multiple Indigenous responses (1.2%), and non-Indigenous (2.7%) [19]. Many community members are employed in a traditional manner utilizing resources of the land (e.g., hunting, fishing, trapping), others in a less traditional manner (e.g., lumbering, tourism, wild rice harvesting), and some are employed through the hospital and schools. The community currently has one elementary school with approximately 200 students from preschool to Grade 6, and one high school serving Grades 7–12 with adult educational programming. Île-à-la-Crosse has a regional hospital with Emergency Services, which includes a health services centre with a total of 29 beds. Other infrastructure of the community includes a Royal Canadian Mounted Police (RCMP) station, a village office, volunteer fire brigade, and a catholic church [46].

### Needs assessment approach

Île-à-la-Crosse shared their vision of integrating digital technology and infrastructure as part of its growth, thus the needs assessment was identified as an appropriate method to provide the formative information necessary to understand what the needs are, including who (i.e., players, partners), and what (i.e., information sources) would need to be involved, what opportunities exist to address the needs, and setting priorities for action with key community stakeholders [47]. As a starting point and rationale for this needs assessment, the community of Île-à-la-Crosse values the potential of technology for improving health communication, information reach, access to resources, and care, and was interested in identifying priorities to begin building digital infrastructure. Given the timing of the COVID-19 pandemic, being responsive to community health needs were key priorities that they wanted to start addressing using a digital platform. This needs assessment facilitated and enabled new conversations around key priorities and next steps.

The evaluation approach was culturally-responsive and included empowerment principles [48–50]. Empowerment evaluation intends to foster self-determination. The empowerment approach [50] involved community members–represented through the Citizen Scientist Advisory Council–engaging in co-production of the evaluation design and implementation by establishing key objectives for the evaluation, informing evaluation questions, building relevant and culturally responsive indicators, developing focus group guides, leading recruitment and data collection, and interpreting results [51]. In this way, the approach incorporated local community and Indigenous Knowledges as well as Western knowledge, in a similar approach to Two-Eyed Seeing [13–15]. Using these needs assessment evaluation results, the community will identify emerging needs and potential application issues, and work with the researchers to continue shaping project development and implementation.

### Two-Eyed Seeing to embed digital platforms

Two-Eyed Seeing as described by Elder Albert Marshall [13, 14], refers to learning to see with the strengths of Indigenous and Western Knowledges. Our engagement and overall approach to working with the community of Île-à-la-Crosse takes a Two-Eyed Seeing lens, from co-conceptualization of solutions, which starts with understanding the needs of the community. All needs are a result of direct Indigenous Knowledge that was provided by the Advisory Council. Indigenous Knowledge is not limited to the knowledge of Elders and Traditional Knowledge Keepers; however, they play a critical role in guiding that knowledge through by providing historical, geographic, and cultural context. Moreover, the Knowledge Keepers can be key decision-makers in the community, and in our case, they were key informants who participated in this needs assessment. Every aspect of needs assessment was dependent on the Advisory Council and Key informants providing the Indigenous Knowledge that the research team needed to tailor digital solutions. As a result, Two-Eyed Seeing approach informed all aspects of the research process.

As we are working to develop, and bring digital platforms and technologies (i.e., Western methods) to address key community priorities, Indigenous Knowledge is central to the overall project. Indigenous Elders, decision-makers, and Advisory Council members are bringing both their historical and lived experience to inform project goals, key priority areas, target groups, and methods. Île-à-la-Crosse is a predominantly Metis community, which differs in culture from other Indigenous communities in Canada—First Nations and Inuit communities. Ceremony is not a key part of community functioning; thus, specific cultural ceremonies were not conducted upon advice of the Advisory Council. Instead, the knowledge of historical issues, challenges, and success stories in the community is considered Indigenous Knowledge for this needs assessment, and more importantly, this Indigenous Knowledge informed the focus areas and next steps for this project. Overall, the spirit of collaboration and co-creation which combined Western research methods/technology with Indigenous Knowledge and expertise is considered Two-Eyed Seeing in this project. This lens was taken at all phases, from the engagement stage to Advisory Council meetings, to planning and executing the needs assessment and next steps.

### Data collection

In order to obtain an in-depth understanding of the key priorities and supports within the community of Île-à-la-Crosse, this needs assessment used a qualitative approach. An environmental scan was conducted in February 2020 of current school and community policies and programs. Published reports, meeting memos, community social media accounts, and the Île-à-la-Crosse website were reviewed for existing policies and programs. The Citizen Scientist Advisory Council identified appropriate data sources for the document review and corroborated which programs and initiatives were currently active in the community.

Qualitative data were collected from key decision-makers and other members within the community. A purposeful convenience sampling approach was employed to identify members of the community who could serve on the Council and participate in focus group discussions. Key decision makers and existing Council members recommended other community members who could join the focus group discussions to provide detailed and relevant information on community priorities, digital infrastructure, supports, and challenges. Two focus groups were conducted by members of the research team in Île-à-la-Crosse with the Council in May 2020. Focus group participants were asked to describe community priorities, supports, and barriers, as well as experience and comfort with digital platforms. Each focus group had four participants, were two-hours in length, and followed an unstructured approach. Three key informant interviews were conducted in Île-à-la-Crosse between February and April 2020. One-hour interviews were conducted one-on-one and followed a semi-structured interview format. The focus groups and key informant interviews were led by the study PI, TRK, and Co-Investigator, JB, who have extensive training and experience with qualitative research methods, particularly in partnership with Indigenous communities. Focus groups and key informant interviews were conducted virtually using Zoom [52]. The key informant interviews and focus groups were audio-recorded and transcribed. All data were aggregated, anonymized, and securely stored in a cloud server. Data are owned by the community. Both the Council and the research team have equal access to the data.

### Data analysis

All documents identified through the environmental scan were reviewed for key themes. A list of existing school and community programs was compiled and organized by theme (i.e., education-focused, nutrition-focused, health-focused, etc.). Follow-up conversations with key informants verified the continued planning and provision of these programs.

Following the 6-step method by Braun and Clarke (2006), a thematic analysis was conducted to systematically identify key topic areas and patterns across discussions [53]. A shortlist of themes was created for the key informant interviews and focus groups, respectively. A manual open coding process was conducted by two reviewers who reached consensus on the final coding manual and themes. Separate analyses were conducted for key informant interviews and focus group discussions; however, findings were synthesized to identify key themes and sub-themes in key priorities for the community, community supports and barriers, as well as digital connectivity and infrastructure needs.

## Needs assessment findings

The needs assessment guiding framework informed specific discussions of key issues in the community of Île-à-la-Crosse. Key informant interviews and focus group discussions commenced by asking about priorities–“what are the key areas of focus for the community?” In all conversations–including a document review of initiatives in Île-à-la-Crosse–health was highlighted as a current priority; hence, questions in the guiding framework were tailored to fit a needs assessment focused on community health. The following five overarching evaluation questions were used to guide the evaluation: i) What are the prominent health issues facing residents of Île-à-la-Crosse?; ii) What supports are currently available to help residents address prominent health issues in the community?; iii) What types of barriers do community members face to accessing services to manage their health?; iv) How is health-related information currently shared in the community?; and v) To what extent are health services and information currently managed digitally/electronically? The evaluation questions were kept broad to capture a range of perspectives. An evaluation matrix linking the proposed evaluation questions to their respective sub-questions, indicators, and data collection tools is outlined in **Table 1**.

**Table 1:** Evaluation matrix of a needs assessment focused on community and digital health.

Original framework question for each topic area	Questions & Sub-questions	Indicators	Data Sources
**Priorities**			
1. What are the key areas of focus for the community?	• What are the prominent health issues facing residents of Île-à-la-Crosse? ○ What are the key health issues among children and youth in the community?	Qualitative feedback from key informant interviews and focus groups	Key informant interviews with the decision makers of the school division and community Unstructured focus groups with the Citizen Scientist Advisory Council
**Current supports**			
1. What supports are currently in place to address this area? 2. How well do the supports currently address this area? 2.1. Are there ways that these supports could be improved?	• What supports are currently available to help residents address prominent health issues in the community? ○ What types of supports are available at the i) school, and ii) community level? ○ What factors influence the use of these supports? ○ What are ways these supports could be adapted to better suit your community’s needs?	Qualitative feedback from key informant interviews and focus groups School programs, school policies, school budget Community Research Conference Executive Summary Document	Key informant interviews with the decision makers of the school division and community Unstructured focus groups with the Citizen Scientist Advisory Council Document review
**Desired supports**			
3. What supports do you need for this area? 3.1. What types of tools or resources could help support this area (e.g., funding, personnel, technology, tailored tools)? 3.2. In what ways could we maximize the reach of new supports (e.g., remote digital engagement)?	• What supports do you wish were available to help residents address prominent health issues in the community? ○ What type of supports would they be? (e.g., resources, infrastructure, programming, policies) ○ In what ways could we enable more people to access the new supports (e.g., remote digital engagement)?	Qualitative feedback from key informant interviews and focus groups School programs, school policies, school budget Community Research Conference Executive Summary Document Community health infrastructure	Key informant interviews with the decision makers of the school division and community Unstructured focus groups with the Citizen Scientist Advisory Council Document review
**Barriers**			
4. Are there any barriers to addressing this priority? 5. What could help overcome this barrier (e.g., funding, personnel, technology, tailored tools)? 5.1. Are there any community members that are disproportionately impacted by this barrier (e.g., youth, Elders)?	• What types of barriers do community members face to accessing services to manage their health? ○ Are there ways to support the community to help them overcome these barriers? ○ Are there specific barriers for children and youth in particular?	Qualitative feedback from key informant interviews and focus groups Community Research Conference Executive Summary Document	Key informant interviews with the decision makers of the school division and community Unstructured focus groups with the Citizen Scientist Advisory Council Document review
**Community engagement**			
6. What are the existing modes of communication in the community (i.e., social media, radio, word-of-mouth)? 6.1. Who is typically part of the conversation on this topic area (i.e., which members of the community)? 6.2. How often do community members connect on this topic or area of interest?	• How is health-related information currently shared in the community? ○ How did you find out (for community members) /communicate (for decision-makers) health information during the COVID-19 pandemic?	Qualitative feedback from key informant interviews and focus groups	Key informant interviews with the decision makers of the school division and community Unstructured focus groups with the Citizen Scientist Advisory Council
**Digital infrastructure and connectivity**			
7. How accessible is i) data, or ii) WiFi in the community? 8. Are there any digital tools currently being used to help address this area? 8.1. If yes, what is the reach of these digital tools (e.g., user groups)? 8.2. If yes, how comfortable are community members with these digital tools (i.e., digital literacy)? 8.3. If not, where or how could a digital platform/tool help with this area? 9. What factors enable/discourage use of digital tools? 10. What are some barriers preventing community members from using digital tools in this area? 10.1. What could facilitate access or use of digital tools in this area?	• All questions from column 1. • To what extent are health services and information currently managed digitally/ electronically? ○ Do you see any benefit of increasing the digital infrastructure for health services in the community? ○ What are some challenges applying digital tools to healthcare in the community?	Qualitative feedback from key informant interviews and focus groups Smartphone use and access in the community WiFi/data connectivity and access	Key informant interviews with the decision makers of the school division and community. Unstructured focus groups with the Citizen Scientist Advisory Council

Feedback on each needs assessment topic area is summarized in the sections below. Sample quotes supporting each of the key topic areas is provided in Table 2.

**Table 2:** Sample quotes supporting needs assessment topic areas.

Key Topic Area	Sample Quotes
**Priorities**	“[There are] pre-existing risks [and] issues like diabetes, blood pressure, and other diseases that the community has dealt with it in the past.” (**KI 1)** “Floods can also be an issue for the community and […] if this data could be implemented on the app that would be great.” (**KI 1)** “…the municipality is taking care of the most vulnerable people, mainly their food and medicine. Food security [is] one of the major issues for the community.” (**KI 1)** “[is] it was possible to access data about [community members’] food needs and to get information on what these needs are?” (**KI 2)** “Mental health issues with the youth is a concern [in the community] during this time.” (**K1 2)** “[Climate change] actually is starting to worry me a lot more now. Comparing last year’s weather to this year, it’s a huge difference […] both in terms of temperature and how it looks outside. Last year we were already getting snow in mid-October and this year we did get our first snowfall around that time, but the snow didn’t stay. It melted away and only really started to stay about hallway through November.” (**FGR1 R1**) “I know some of my family talked [about] it. Because based last year during December versus this year early December where this year there wasn’t that deep snow, meanwhile last year we already had snow going up to our knees at the time. I think when I do think about [climate change], then it’s definitely stresses me out. (**FG1 R2)** “[Climate change] does stress me out. Especially, last year, I saw a bear out that was not supposed to be out.It was way too early and there was still snow and it looked confused and I was like this is actually happening. . .this is climate change. Especially with this year, the kind of messed up weather we were having was warm and all of a sudden, it got extremely cold. It’s just kind of all over the place” **(FG2 R1)** “I feel like the biggest concern with all that is our main road. We have only one road out of here in summer. Like there is one road in, and one road out. So that would be the main concern of how do we get out, how do people come in, like…. and that’s probably the biggest concern.” **(FG2 R2)**
**Current supports**	“…wellness can mean different things to different people […] For example, for some individuals, wellness means spirituality, which is associated with language and culture, while for others it means being physically, mentally, emotionally and spiritually healthy.” **(KI 2)** “Through extensive discussions regarding wellness and what wellness meant to each individual, the following themes emerged as components to be included in the wellness model: 1. Healthy parenting; 2. Healthy youth; 3. Healthy communities; 4. Elders; 5. Healing towards wellness; 6. Food sovereignty” **(Northern Village of Île-à-la-Crosse–Community Research Conference Executive Summary Document)** “A significant portion of the gathering was dedicated to the importance of Elders, who are seen as Knowledge Holders who are best positioned to revive and reinvigorate the sorely missing spiritual and cultural aspects of the community. Elders have both enormous responsibility and immense capacity to share traditional knowledge and teachings. Therefore, the Elders’ Lodge being part of and at the core of the initiative will provide many opportunities for community and intergenerational knowledge transmission.” **(Northern Village of Île-à-la-Crosse–Community Research Conference Executive Summary Document)** “The community prioritizes food sovereignty and initiatives such as the greenhouses for growing vegetables and fruits. There are after-school programs (traditional food education: preparing and cooking bannock, duck, fish and other traditional foods), and community land-based activities (e.g., berry picking led by Elders), which heighten awareness of food security and sovereignty, and encourage Youth-Elder bonding and intergenerational knowledge transmission.” **(Northern Village of Île-à-la-Crosse–Community Research Conference Executive Summary Document)** “I had a class that was heavily focussed around the greenhouse last year, but besides that I haven’t really gone to the greenhouse much this school year.” (**FG1 R1)** “We were helping around in the greenhouse a lot. Planting new crops, harvesting them. We also had our own little side crops that we did for a class project where we each planted our own crops and grew them and wrote down the different growth stages on it. […] Yeah, I enjoyed it.” **(FG1 R2)** “[The greenhouse program] is still ongoing and it’s beautiful in there, and there is a lot of plants. And we have planted a lot of those plants and we harvests a lot of those plants as well. And we do that to take home a lot of the food that we grow and collect from the green house so that we could take it home to… you know, cut it up for supper and stuff like that, and sometimes our projects are to take pictures and show what we’ve made out of the stuff that we have taken home. So, it’s still pretty good program being ran by the school.” **(FG2 R1)**
**Desired supports**	“Looking to the future, the overall improvement of family wellness, and indirectly community wellness, will be viewed through the holistic lens of healthy parenting, with interventions focused on mental wellness and addressing substance misuse.” **(Northern Village of Île-à-la-Crosse–Community Research Conference Executive Summary Document)** **“**With various existing programs focusing on youth wellness in the community, nuanced changes were needed, especially by incorporating Elders and the Elders’ Lodge. The consensus was that Île-à-la-Crosse needed well-nourished youth who are educated in land-based activities and enjoy rich Elder-Youth bonding.” **(Northern Village of Île-à-la-Crosse–Community Research Conference Executive Summary Document)** “Traditional language revitalization by and through youth and young adults was prioritized as an urgent need and viewed as the essence of the wellness model. Additionally, the community expressed a commitment to a respectful relationship with and stewardship of the environment through programs that re-develop traditional family activities. Furthermore, addressing the root causes of trauma (e.g., colonialism, oppression, stigma) through mental wellness and addiction programming that promote a supportive and inclusive community.” **(Northern Village of Île-à-la-Crosse–Community Research Conference Executive Summary Document)**
**Barriers**	“I was turned down when I went to get glasses because I’m from the northwest” (**FG1 R4**) “The community has to fight against different types of racism. Young people are invaluable in this process.” (**KI 2**) “[For COVID] we need to address household risk, especially because of overcrowding.” (**FG1 R3**) “Some of the community members from various healthcare and educational backgrounds pointed out the potential for illness and addiction in the community. They noted that there continues to be stigma associated with community programs addressing these issues, which inhibit participation and healing. Going back to the root causes of dysfunction and understanding the history of the community and the oppression and colonialism it has experienced will help individuals and the community to embark on healing journeys towards achieving wellness.” **(Northern Village of Île-à-la-Crosse–Community Research Conference Executive Summary Document)**
**Community engagement**	“…mainly we use radio and social media–like Facebook–to share information in the community” (**KI 3**) “I feel like this app is going to be very positive […] and it’s also [the Internet is] just full of lies, this app will really connect you to reality…” (**FG2 R3)** “Every so often I do [listen to the radio], but not that often. Only whenever it’s on and I’m too lazy to change it… I would say [we get news from] a mixture of people spreading it around and radio station I would say. A mixture of that.” **(FG1 R1)** “I think Facebook is definitely a way people can check [for COVID information]… I think the radio is great for sharing information but I think that there is not a lot of people that…like there aren’t many teens listening to the radio” (**FG1 R3)**
**Digital access and connectivity**	“I look forward to the project because [we] need to embrace technology moving forward. There have been some technological advancements being used at the nursing program here, and technology will help with the issues that our Northern communities face.” (**KI 3)** “We need to have assistance for the Elders when it comes to explaining how to use the applications.” (**FG1 R1)** “I don’t check my email often I do I do for work, but that’s like three times a week. I don’t use it that often. I think through the app directly and notifications, I’ll be the best.” (**FG2 R2)** “[Internet speed] is good. It depends what provider you are using and where you live in town.” (**FG1 R2)** “Where you live where you come into town, that area is where it cuts off every so often and it’s more slow around that area. But when you get more towards town and near the ICSI building and you use the actual ICSI internet provider, that’s where it tends to be a lot smoother.” **(FG2 R1)** “…But with Sasktel its mostly a smooth performance all around town here.” (**FG2 R2)** “[For sharing information], I would say just texting each other honestly…because even on snapchat, that’s another way a lot of, I know my peers connect with each other. I stay in touch with a lot of people outside of this community and with friends in Saskatoon, and other places.” **(FG1 R1)** “I think the main way the kids and teenagers these days are connecting is through texting and snapchat. Even though I don’t have snapchat, I think that a lot of people, like 90% of people in the school, the main way of connecting is probably through snapchat.” **(FG1 R2)** “We have good cell service in the village…we know all of them use [smartphones] and we need to educate them about data safety. This is important for building apps for [community] problems.” (**KI 2**)

FG–Focus Group; R–Respondent; KI–Key Informant

### Key priorities

**Fig 2:**
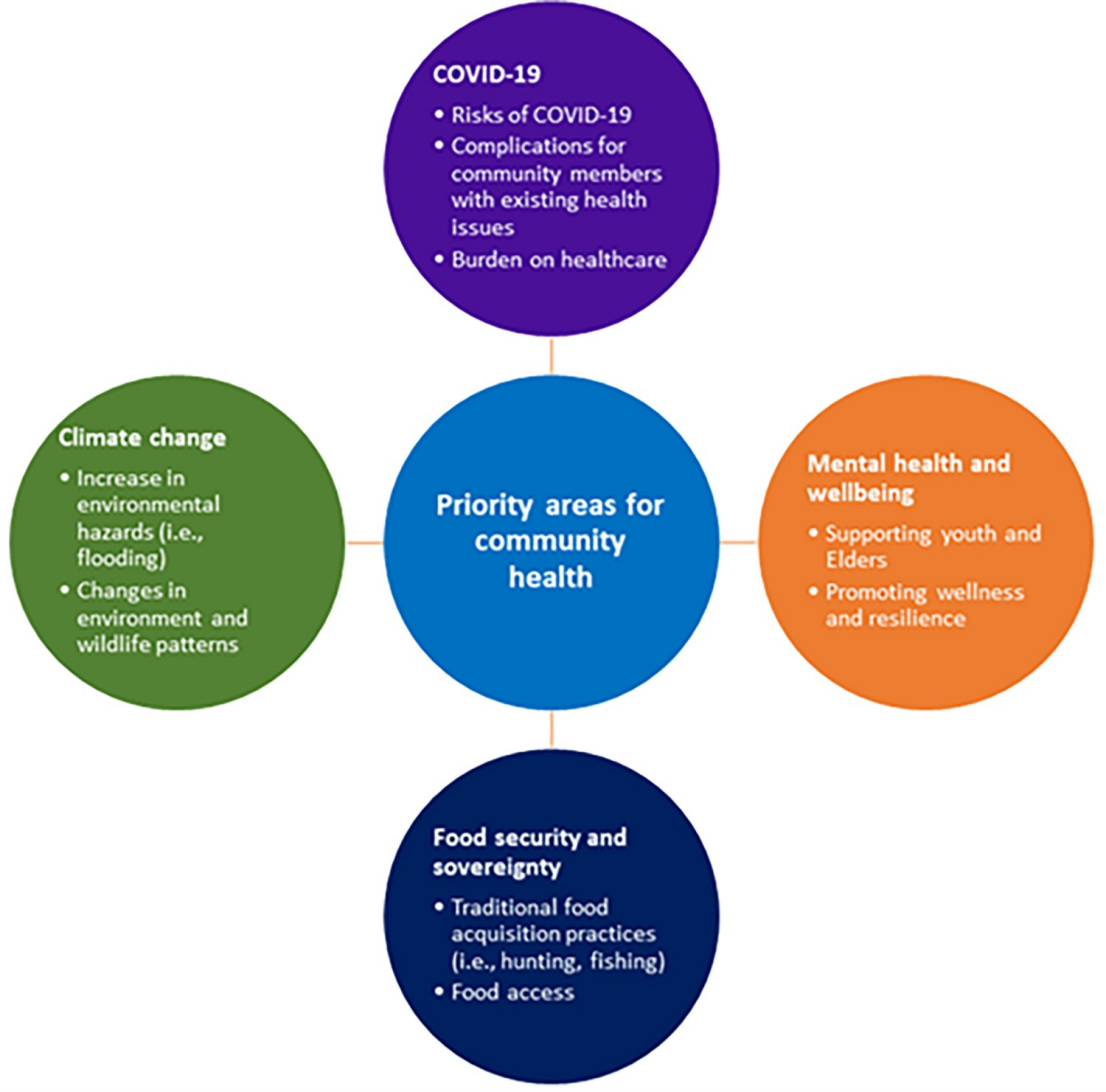
Summary of key health priorities.

Four priorities were identified through the focus groups, key informant interviews, and document review (Fig 2). Given the timing of the discussion, the primary issue of concern was the COVID-19 pandemic. Many community members were worried about contracting the virus, and the risk it posed to Elders in the community. Of greater concern, however, was how COVID-19 exacerbated many existing health concerns including diabetes and hypertension in the community. For example, routine procedures were postponed and community members with other health conditions were not receiving routine healthcare during the height of the pandemic. The St. Joseph’s Hospital and Health Centre services Île-à-la-Crosse and bordering communities, hence maintaining capacity for COVID-19 patients was a priority. COVID-19 exposed existing barriers in the healthcare system which are described in greater detail in the barriers to community health section.

Another priority discussed by many community members was climate change and the environment. Community members noted that changes in wildlife patterns, land use, and early winter ice road thaw were areas of concern, particularly due to the impact these factors have on traditional food acquisition practices (i.e., hunting) and food access. For instance, the geographic location of Île-à-la-Crosse is surrounded by a lake, and the main highway which connects the community to the land has experienced increased flooding in the past few years.

In addition to posing immediate danger to community members, food security and sovereignty are also closely linked to road access. While the community produces some of its own food through the local fishery and greenhouses, Île-à-la-Crosse is still dependent on a food supply from the south (i.e., Saskatoon). During COVID-19, food access was further restricted due to limited transport and delivery of food products, which increased the risk of food insecurity for community members. Food insecurity was believed to be of bigger concern for Elders in the community compared to younger members. Younger community members expressed having the ability to source their own food in a variety of capacities (e.g., fishing in the lake), whereas Elders rely more heavily on community resources and support (e.g., grocery stores, friends, and family).

Community members also discussed issues surrounding mental health and wellbeing. This topic was of particular concern for youth and Elders in the community. Community members discussed the importance of identifying covert racism (vs. discrimination) that exists within health services that exacerbated mental health issues and care, as well as developing coping strategies, resilience, and supports to prevent mental health crises. Key informants emphasized the need to minimize the stigma around mental health and focus on holistic wellbeing as they work to develop strategies to improve community wellness.

### Community health supports

Île-à-la-Crosse has been working on developing supports to improve community health through various initiatives. A document review identified a community-specific wellness model which has informed program development and planning over the past few years. The key components of the Île-à-la-Crosse wellness model are: i) healthy parenting; ii) healthy youth; iii) healthy communities; iv) Elders; v) healing towards wellness; and vi) food sovereignty. The Elders Lodge in the community provides support for holistic wellbeing by promoting intergenerational knowledge transmission, guidance to youth and community members, as well as land-based activities which improve bonding, cultural awareness, and mental and spiritual well-being among community members. The Elders Lodge hosts both drop-in and organized events.

Several initiatives have been developed to support food sovereignty in the community, including a greenhouse program where fruits and vegetables are grown and shared locally. This program is run in partnership with the school to increase food knowledge and skills among youth. In addition, after-school programs including traditional food education (i.e., cooking classes) and land-based activities (i.e., berry picking) led by Elders support the goals of the wellness model. The community is currently working on developing additional programs dedicated to improving mental wellness among adults, youth, and Elders.

### Barriers to community health

When key informants were asked to identify barriers to community health, they described delays in access to timely health information. For example, daily COVID-19 tests conducted at the regional health centre in Île-à-la-Crosse were relayed to the provincial health authority; however, information about the total number of COVID-19 cases could take up to one week to be sent back to the community. This time lag restricted community decision-makers’ ability to enact timely policy (i.e., contact tracing) and rapidly respond to managing cases.

A second barrier that was raised by community members was a delay in access to timely healthcare. The Île-à-la-Crosse hospital is a regional health service centre serving the community as well as surrounding areas. Community members noted that the load often exceeded the capacity of the single hospital, and some patients and procedures were relocated to hospitals and clinics in the larger city of Saskatoon, Saskatchewan. This was reported to be challenging for many community members as it was associated with longer wait times, long commutes, and sometimes required time off work. Many of these challenges were exacerbated during the COVID-19 pandemic. As a result of the pandemic, many medical centres and hospitals postponed routine and elective medical procedures in an attempt to accommodate the overwhelming influx of patients who contracted COVID-19. In addition, community members were advised to avoid spending time in health centres to limit risk of exposure to the virus. These COVID-related changes further delayed access to timely healthcare for many community members of Île-à-la-Crosse.

Several community members reported experiencing institutional racism in healthcare and social service settings outside of Île-à-la-Crosse. This was particularly exacerbated during the COVID-19 movement restrictions, where community members faced significant difficulties in accessing services and care in larger urban centres, and experienced further discrimination due to the stigma of COVID-19-related rumours about communities in the north.

Lastly, community members discussed a lack of awareness about some health topics, including where and how to access reliable health information. Some community members attributed this lack of awareness to a general distrust in government health information due to a history of colonialism and exploitation in Canada, which likely contributed to increased misinformation about COVID-19 risk and spread.

### Health communication

The primary modes of communication within Île-à-la-Crosse are radio and social media. These platforms were used throughout the pandemic to communicate health information about COVID-19 case counts and trends. Community members also reported obtaining health information from healthcare practitioners (i.e., for those already visiting a healthcare provider), Elders, and the internet. Key informants indicated an interest in improving digital infrastructure to enable sharing of timely and accurate health information with community members and minimize misinformation. Key informants also reported room for improvement in the community’s digital health infrastructure, particularly in improving timely communication with community members, and to inform decision-making in crisis situations.

### Digital infrastructure and connectivity

Île-à-la-Crosse has its own cell tower which offers reliable access to cellular data. The community also has access to internet via the provincial internet provider–SaskTel, as well as a local internet provider—Île-à-la-Crosse Communications Society Inc. Key informants and community members confirmed that most individuals above 13 years of age have access to smartphones, and that these mobile devices are the primary mode of internet access. However, it was unclear whether everyone who owns smartphones also has consistent data plans or home internet connections. Key informants described the great potential of digital devices like smartphones to increase the speed and accuracy of information sharing. Discussions with both key informants and community members suggested the need for a community-specific app or platform which could provide timely health information that was tailored to the community’s needs.

Community members noted that expanding digital infrastructure had to be paired with efforts to improve digital literacy–particularly as it relates to data security, privacy, and online misinformation. A separate initiative was discussed which could work to improve digital literacy among youth and Elders, as this would improve both the uptake of digital health platforms, as well as their usefulness and application. Key informants discussed the importance of building digital infrastructure that would enable data sovereignty, self-governance, and determination. The key informants, who are also primary decision-makers in the community, described opportunities for ethical development of digital platforms that would ensure that data is owned by the community.

## Discussion

Needs assessments are commonly the first step in understanding specific community needs, [27, 28]; however, few evaluation frameworks provide practical guidance on how to engage communities in needs assessments [41]. This paper provides a step-by-step guide for conducting needs assessments in collaboration with communities in the digital age. Using the series of questions outlined in the Guiding Framework, researchers and evaluators can gain an in-depth understanding of a community’s priorities, needs, existing capacity, and relevant solutions.

The Guiding Framework was critical to establishing a partnership with the community of Île-à-la-Crosse, as it enabled the research team to obtain detailed insight into their priorities–in this case, community health–as well as community capacity. Taking a Two-Eyed Seeing approach [15], conversations with the community highlighted strengths of Western digital technology and the diversity of Indigenous Knowledges for addressing priorities [13]. This approach was also important to establishing trust and respect for the variety of perspectives that could be used to address community priorities. The resulting partnership also enabled the conceptualization of tangible action items that were aligned with current and future priorities–a key factor in the sustainability and feasibility of community-based initiatives [4–8, 54].

### Challenges and opportunities for using digital platforms for priorities identified by needs assessment

Many rural and remote communities face similar challenges and share common priorities with Île-à-la-Crosse. For example, resource and service access, including food and other essential supplies, healthcare, and internet connectivity are issues faced by many rural and remote communities across Canada [55–60]. Key informants and community members from our partner community corroborated these access issues, particularly in relation to public health. Given the potential for digital technology to bridge access gaps, it has become pertinent to invest in digital infrastructure and platform development.

Research has shown that in many rural and remote communities, smartphone ownership is not the limiting factor–it is internet inequity, which is defined as differential internet access based on wealth, location (urban, rural, or remote), gender, age, or ethnicity [61]. The United Nations has declared internet access a human right [10], which makes it imperative to develop digital infrastructure such as internet connectivity to improve digital accessibility. Île-à-la-Crosse has its own cell tower which offers reliable access to cellular data. The community of Île-à-la-Crosse also has access to consistent and dedicated internet service through a provincial internet provider and local internet provider. The needs assessment showed that the universality of smartphone ownership combined with good internet connectivity lays the foundation for the development of tailored, culturally appropriate digital health platforms in communities like Île-à-la-Crosse.

In particular, the needs assessment revealed that smartphone apps, which most citizens are well-versed with, can be used to provide local services and access to resources. For example, a locally developed app can connect the Mayor’s office with community members in real-time to provide updates on COVID-19 outbreaks. Apps also have the potential to connect communities to resources within and outside of the community [35, 57]. For example, advanced artificial intelligence algorithms can be used to anticipate community needs prior to urgent crises like COVID-19, environmental disasters, or food crises [35, 62–65]. To date, the issue has not been the lack of technology or ability to bridge this gap for rural and remote communities. Instead, larger systemic inequities have limited our ability to co-create local solutions for global problems by decentralizing technology that is widely available [35, 66], which highlights upstream inequities in developing digital platforms.

### Recommendations for inclusive digital needs assessments

Given the widespread adoption of digital technology, digital platforms can provide rich data to identify and address community crises [2, 3, 35]. Importantly, co-created digital platforms can be used to share knowledge in real-time with community members and other stakeholders to enable remote engagement, which is especially important during crisis situations such as a pandemic [2, 3, 35]. As we implement creative digital platforms in varied programs or research projects, we must also integrate this digital perspective into the evaluation process. Research and evaluation literature has well established approaches to needs assessment evaluations [29, 42, 67]; however, in the 21st century, we need to account for the use and application of digital platforms in community-focused initiatives. To identify how and where digital platforms can play a role in addressing community priorities, we propose several recommendations for inclusive community-based needs assessments.

First, at the crux of all community-based needs assessments is relationships. A relationship built on respect, reciprocity, mutual understanding, and prioritizing the needs and vision of communities is essential for sustainable impact. The First Nations OCAP® principles [68] informed conversations between the research team and community about data ownership and control. These principles include ownership of knowledge and data, control over all aspects of research, access to information about one’s own community, and possession or control of data [68]. The OCAP® principles ensure First Nations and other Indigenous Peoples the right to their own information, and also reflect commitments to use and share information in a way that maximizes the benefit to a community, while minimizing harm. Some communities may choose to lead a project, or work closely in collaboration with experts for specific projects. Irrespective of the project dynamics, needs assessments rely on detailed information and context about a community for a project to succeed.

Second, it is important for researchers and evaluators to gain an understanding of the current digital infrastructure and connectivity in the community. The needs assessment framework (Fig 1) includes relevant questions for identifying data and WIFI access in a community, penetration of digital devices, and existing digital infrastructure. Even for community-based initiatives that are not focused on a digital platforms, digital technologies will inevitably be a part of the solution, a barrier, or both. Hence the digital landscape has become part of the context that we must capture and understand in a needs assessment to better design and develop programming, policies, and other initiatives.

Third, it is important to ask the question of where and how a digital tool or platform could help. Are there gaps that digital platforms can help address or fill? In rural and remote communities, in particular, digital platforms can provide access to real-time information and services not otherwise available. For example, Telehealth [69, 70] in the Canadian north offers citizens access to essential healthcare services, including video appointments with medical specialists. Prior to Telehealth, many residents would need to fly into bigger cities in the nearest province to access health care [55].

Lastly, an understanding of the broader context which affects a community’s ability to adopt digital platforms is critical to the success of digital initiatives. This includes, but is not limited to, capturing data on socioeconomic status and the accessibility of internet-connected digital devices. Digital platforms should help to bridge the divide in resource, service, and information access–not widen the gap. For some communities, this may require working on building digital infrastructure and obtaining dedicated funds to expand access prior to implementing digital initiatives. In addition, digital literacy cannot be taken for granted. Digital literacy refers to individuals’ ability to not only use digital devices, but according to Eshet-Alkalai [71], “includes a large variety of complex cognitive, motor, sociological, and emotional skills, which users need in order to function effectively in digital environments.” In its simplest form, digital literacy may include the ability to navigate digital platforms, download apps, and communicate electronically. Other more specific skills include ability to read and understand instructions, terms and services, as well as data privacy and security statements [72–74] As part of a needs assessment, identifying digital literacy within a community is an important step to safe, ethical, and relevant digital tool development.

Considering the challenges, immense potential, and learnings from applying the Guiding Framework, a tailored digital platform was conceptualized called Sakitawak Health.

### Development of Sakitawak Health

Sakitawak Health is a culturally-responsive digital epidemiological platform to monitor, mitigate, and manage COVID-19 outbreaks. The needs assessment concluded that digital platforms can be used for emerging or other existing population health crises within Île-à-la-Crosse and potentially other Indigenous communities. Moreover, to co-create digital platforms, the Île-à-la-Crosse Citizen Scientist Advisory Council identified key features to embed in CO-Away, including free virtual care for citizens via a smartphone app at the frontend, and access to anonymized community data on the backend for decision-makers.

The app will provide three key precision medicine services that are specific to each citizen: 1) continuous risk assessment of COVID-19 infection; 2) evidence-based public health communication; and 3) citizen reporting of food availability, access to public services, and COVID-19 symptoms and test results. These culturally-responsive features have been co-created with Métis decision-makers in Île-à-la-Crosse based on imminent community needs and preferences. CO-Away will enable real-time data collection through continuous citizen engagement to inform municipal jurisdictional policies.

There are three guiding principles for developing Sakitawak Health: I) Citizen empowerment and data ownership: Active engagement is enabled through app features such as visualizing community risk. More importantly, the community owns the data to ensure data sovereignty; II) Privacy: Utilizing a cutting-edge methodology called federated machine learning, we will develop artificial intelligence algorithms that stores sensitive data such as participant location on mobile devices itself (i.e., sensitive data are not stored in external servers); III) Security and scalability: The backend server will be located in Cloud in Canada, which allows for horizontal and vertical scalability (i.e., the potential for developing multiple frontend apps and decision-making dashboards).

### Recognizing the importance of data sovereignty and Indigenous self-governance

Data sovereignty and social justice are important aspects of community-based work, particularly for communities that have experienced discrimination or systemic inequities [2, 75]. Data sovereignty refers to meaningful control and ownership of one’s data [76]. For Indigenous communities in Canada, self-determination and self-governance are of paramount importance given the colonial history of oppression, trauma, and disenfranchisement [77], and data sovereignty and ownership of digital platforms can promote that independence. In conducting digital community-based needs assessments, the application of a Two-Eyed Seeing lens enables us to leverage strengths of both Indigenous and Western Ways of Knowing to help focus on key priorities and develop solutions.

The engagement and overall approach to working with the community of Île-à-la-Crosse applied a Two-Eyed Seeing lens. In the needs assessment with Île-à-la-Crosse, Two-Eyed Seeing involved incorporation of Métis Knowledge during team engagements, which ensured that any digital platforms developed would incorporate Indigenous Knowledge to promote data sovereignty. All priorities identified within this manuscript are a result of direct Indigenous Knowledge that was provided by the Council. Indigenous Knowledge is not limited to the knowledge of Elders and Traditional Knowledge Keepers; however, they play a critical role in guiding that knowledge through by providing historical, geographic, and cultural context. Discussions with Île-à-la-Crosse about data sovereignty centered around citizen ownership of data, community access, and ensuring data privacy and security. The ultimate goal of this approach to data sovereignty is to facilitate decreased dependence on external systems and use digital solutions for Indigenous self-determination and self-governance.

### Next steps

The needs assessment represents the first phase of a larger evaluation strategy to develop and implement culturally appropriate digital platforms for community health. Phase 1 involved identifying core health priorities and desired supports in the community of Île-à-la-Crosse. Based on the needs assessment findings, Phase 2 of this project will involve the development of tailored digital health platforms and programming to support digital literacy. As part of Phase 2, digital literacy programs and tailored digital health platforms will be pilot tested and adapted prior to their implementation. In Phase 3, a process evaluation will be conducted to assess the reach, uptake, and use of digital health platforms and digital literacy programming. Integrated knowledge translation will be conducted during all phases to ensure continuous feedback, communication, and knowledge sharing with all relevant stakeholder groups.

## Conclusions

Needs assessments can facilitate important conversations in community-based research and evaluation to learn about key priorities, challenges, and opportunities for growth. The Guiding Framework for Community-Based Needs Assessments to Embed Digital Platforms details a step-by-step approach to begin a conversation with communities to better understand their needs, and to tailor research and evaluation projects focused on embedding digital platforms. In Île-à-la-Crosse, the needs assessment framework has propelled the launch of a timely, community-engaged digital initiative to address key priorities, starting with COVID-19. Overall, tailored platforms can help bridge existing gaps in resource, program, and service access in Indigenous communities, irrespective of their location across the world.

## Supporting information

S1 File (DOCX)
