# Correction: A guiding framework for needs assessment evaluations to embed digital platforms in partnership with Indigenous communities

**DOI:** 10.1371/journal.pone.0305487

**Published:** 2024-06-10

**Authors:** Jasmin Bhawra, M. Claire Buchan, Brenda Green, Kelly Skinner, Tarun Reddy Katapally

The Fig 1 is low resolution. Please see the correct Fig 1 here.

**Fig 1:**
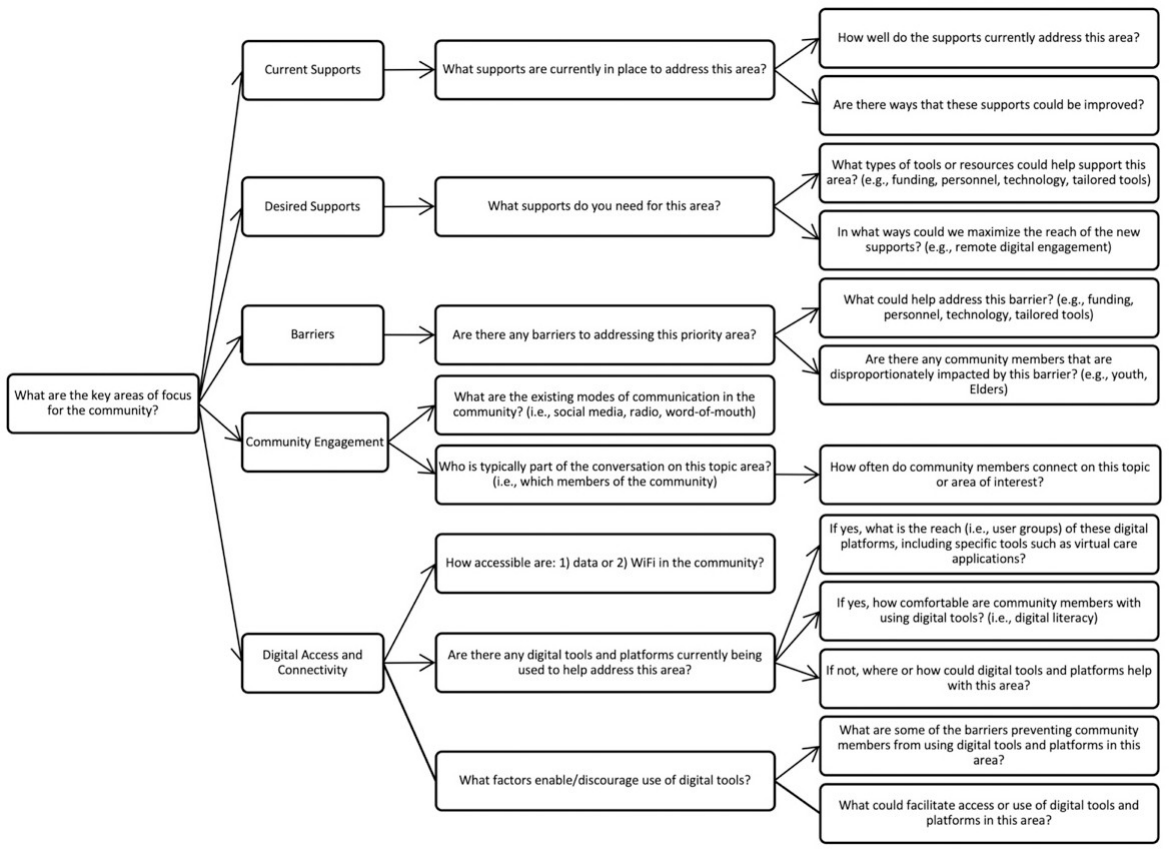
A guiding framework for community-based needs assessments to embed digital platforms.
